# Etymologia: *Aedes aegypti*

**DOI:** 10.3201/eid2210.ET2210

**Published:** 2016-10

**Keywords:** etymologia, Aedes aegypti, Culex aegypti, mosquitoes, human diseases, chikungunya, dengue, yellow fever, Zika, Fredrik Hasselqvist

## *Aedes aegypti* [a-eʹdēz a-jipʹtē]

In 1757, Fredrik Hasselqvist (a protégé of Carl Linnaeus) first described a mosquito collected in Egypt as *Culex* (Latin for “gnat”) *aegypti *(Figure), noting as the most salient feature the “glistening white” rings on the legs. *Aedes* (Greek for “unpleasant”) *aegypti* is the principal vector of several human diseases, including chikungunya, dengue, yellow fever, and Zika. Yellow fever virus was among the first human viral pathogens to be discovered, and the US Army Yellow Fever Commission’s work showing that *Ae. aegypti* (also known as the “yellow fever mosquito”) was the principal vector remains one of the cornerstones of medical virology and tropical medicine.

**Figure:**
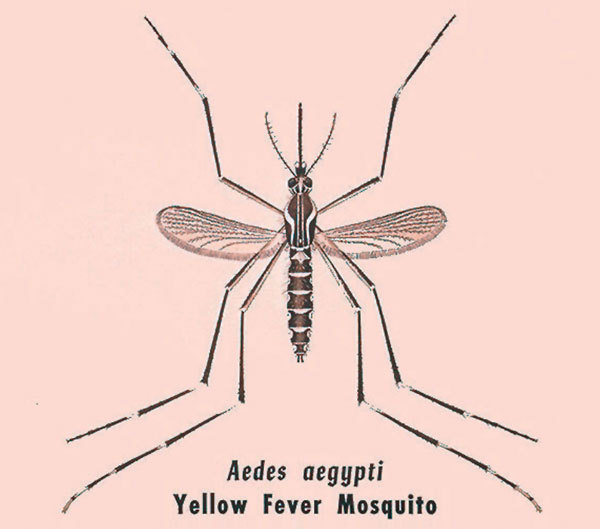
Illustration of *Aedes aegypti* adult mosquito, vector of yellow fever. CDC/ James M. Stewart

*Ae. aegypti* arrived in the New World shortly after Europeans, transported on ships, where conditions selected for the anthropophilic *Ae. aegypti* subsp. *aegypti*. (Forest-breeding zoophagous *Ae. aegypti* subsp. *formosus* are still found in sub-Saharan Africa.) From the New World, *Ae. aegypti* spread across the Pacific to Asia and Australia.
